# Recent advances in hyaluronic acid based therapy for osteoarthritis

**DOI:** 10.1186/s40169-017-0180-3

**Published:** 2018-02-16

**Authors:** Steven Bowman, Mohamed E. Awad, Mark W. Hamrick, Monte Hunter, Sadanand Fulzele

**Affiliations:** 1 Department of Orthopedics, Augusta University, 30904 Augusta, Georgia; 2 Department of Oral Biology, Augusta University, Augusta, Georgia; 3 Department of Cell Biology and Anatomy, Augusta University, Augusta, Georgia; 4 Institute of Regenerative and Reparative Medicine, Augusta University, Augusta, Georgia

**Keywords:** Osteoarthritis, Hyaluronic acid (HA), Treatment, Tissue engineering

## Abstract

Osteoarthritis is a debilitating disease that has increased in prevalence across the world due to the aging population. Currently, physicians use a plethora of treatment strategies to try and slow down the progression of the disease, but none have been shown to ubiquitously treat and cure the disease. One of the strategies uses the high molecular weight molecule hyaluronic acid as either an injectable or oral supplement for treatment. Hyaluronic acid (HA) is a relatively new treatment that has shown varied results through several clinical trials. It can be used as a scaffold for engineering new treatments and several new preparations have just been added to the market. A comprehensive search was conducted through several search databases according our inclusion and exclusion criteria. This review included 44 prospective clinical trial investigating the feasibility and efficacy of HA injection for knee, hip, and ankle osteoarthritis. This review will take a closer look at hyaluronic acid and its properties, as well clinical effectiveness and future options.

## Introduction

Osteoarthritis (OA) is a degenerative joint disease that frequently affects the hands and weight bearing joints of the body [1]. In United States, 52.5 million adults have been diagnosed with osteoarthritis according to data analyzed between 2010 and 2012 in the National Health Interview Survey (NHIS) [2]. In addition, OA is considered as one of the main causes of functional disability in (estimated) 22.7 million US adults [3]. The patient with OA is suffering not only from the persistent pain, stiffness and limited mobility. However, it also directly affects their quality of life with physical and/or mental co-morbidity [4]. OA substantially increases health care expenditures which is estimated around $ 128 billion [5]. When considering productivity loss due to OA, estimates are between 0.25 and 0.50% of the Gross Domestic Product (GDP) [5].

Osteoarthritis (OA) is poorly understood because of its vast complexity and interplay of various biological factors such as: genetic alterations, sex hormone deficit, and aging [6]. Many recent evidence has focused on molecular markers that implicated in the stress-induced senescent state of chondrocytes [7]. The term “Chondrosenescence” has been currently used to describe the age-dependent deterioration of chondrocyte function [8]. The therapeutic approaches for OA are limited because of its complex pathophysiology. According to the Osteoarthritis Research Society International (OARSI) Guidelines and recommendations for OA management, a core set of evidence based-modalities of therapy has been established [9]. These modalities included non-pharmacological such as patient education and awareness, physical exercise and rehabilitation aids. The pharmacological modalities vary from prescription of acetaminophen, non-selective NSAIDs (Nonsteroidal anti-inflammatory drugs) and selective COX-2 inhibitors agents and even opioid prescription. NSAIDs are the most prescribed agents for OA [10]. Despite NSAIDs established effectiveness in relieving the pain with OA its long term use is associated with potential harmful adverse effects. In addition, there is a wide heterogeneity in their personalized response because of the pharmacogenomics interactions [11]. The other potential non-operative therapeutic methods are chondroitin sulfate, glucosamine, and intra-articular injections of visco-supplements, corticosteroids, or blood-derived products [9]. However, there is a controversy about their complete efficacy and long-term safety in improving the patients symptomatically [12]. Remarkably, physical therapy such as mind–body exercise, strength training exercises and aerobic exercises has all shown some promising results in improving the OA prognosis as long as the patients are consistently compliant with their physical therapy regimen [13–15]. The nutritional supplements such as dimethyl sulphoxide (DMSO) and methylsulfonylmethane (MSM, present in green plants, fruits and vegetables) have been tried with limited success [16].

Over the last decades, there is an ongoing trend to use Intra-articular injections of either corticosteroids, analgesics/anti-inflammatory drugs, polymerized collagen, anti-cytokine drugs, or hyaluronic acid as alternative modalities to maximize the topical effect and minimize the systemic adverse effects [17]. Each injection has been shown to lower the pain in patients in some form, though hyaluronic acid treatments seem to be the safest and last the longest [17]. This review article highlights the current advances of Hyaluronic acid based therapy for osteoarthritic patients.

**Fig. 1:**
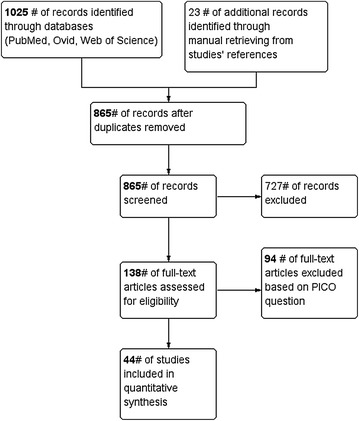
Flow chart showing study identification, inclusion, and exclusion

The literature search was performed through several search databases such as PubMed, Ovid via Medline, and Web of science using wide-spectrum keywords: Hyaluronic acid, hyaluronate injection, visco-supplements; intraarticular; knee, ankle, hip; osteoarthritis, cartilage degeneration. All prospective randomized controlled and retrospective observational cohort trials investigating the efficacy and safety of intra-articular injection of HA were considered for inclusion (Fig. 1).

## Hyaluronic acid physiology in synovial fluid of joint

Hyaluronate is a high molecular weight, ubiquitous molecule that naturally occurs within the cartilage and synovial fluid. It is composed of alternating *N*-acetyl-d-glucosamine and d-glucuronic acid residues attached by β(1–4) and β(1–3) bonds with molecular mass ranging from 6500 to 10,900 kDa [18]. Its rheological characteristics involved in the main function of synovial fluid to serve as a lubricant, scavenger for free radicals, and for the regulation of cellular activities such as binding of proteins [19]. Its functions in the joint include lubrication, serving as a space filler to allow the joint to stay open, and the regulation of cellular activities such as binding of proteins [19]. During the progression of OA, the endogenous HA in the joint is depolymerized from being of a high molecular weight (6500–10,900 kDa) into a lower molecular weight (2700–4500 kDa), which consequently diminishes the mechanical and viscoelastic properties of the synovial fluid in the affected joint [18, 20]. Thus, exogenous HA injections have been clinically used to mitigate the macerated functions of the depolymerized endogenous HA of OA patients [20]. Although the exogenous HA does not restore and replace the full properties and activities of the depolymerized endogenous HA of the synovial fluid but it may induce satisfactory pain relief via several mechanisms [20]. These mechanisms include synthesis of proteoglycan and/or glycosaminoglycan, anti-inflammatory effect, and viscoelasticity maintenance [20]. Nevertheless, there is a clear heterogeneity in the Therapeutic trajectory for OA patients following HA injections. As some studies reporting an overall beneficial effect while others report that there is only a small benefit [21].

One of the potential reasons for the variable effect of HA treatments on OA patients is levels of hyaluronidases in a patient’s synovial fluid. Hyaluronidases are a family of enzymes that degrade hyaluronic acid through cleaving the β(1–4) linkages of HA, fracturing the large molecule into smaller pieces before degrading it [22].

## Hyaluronic acid and its preparations for treatment of OA

HA is being administrated into OA patients via two main ways either oral administration or local injection [23, 24]. Several preparations of injectable HA used for clinical use include Synvisc^®^ and Synvisc-One^®^ (Genzyme); Gel-One^®^ (Zimmer); Hyalgan^®^ (Fidia); Supartz FX™ (Bioventus); Orthovisc^®^ (Anika); Euflexxa^®^, previously named Nuflexxa (Savient); Monovisc^®^ (Anika Therapeutics); and Gel-Syn™ (Institut Biochimique SA) [25]. Each product differs in many characteristics, including source (animal versus bacterial bio-fermentation using modified organisms), mean molecular weight ranging from (500 to 6000 kDa), distribution of molecular weight, molecular structure (linear, cross-linked or both), method of crosslinking, concentration (0.8–30 mg/mL), volume of injection (0.5–6.0 mL), and posology [26]. Although Animal source of HA (rooster combs) was considered as a traditional source for many years. However, many investigations have been performed to develop alternatives for obtaining HA, such as bio-fermentation using genetically modified organisms. This modified bacterial source is currently used as the main source as it’s associated with lower costs and less side effects [27, 28].

Table 1 includes the characteristics of most common injectable hyaluronic acid products that are already approved by Food and Drug administration (FDA).

**Table 1:** Clinical studies evaluating the efficacy of injectable HA in osteoarthritic patients

Authors	Years	Study type	Joint	Patient characteristics		HA injection characteristics				Outcome	
				Number	Mean age	HA brand	HA molecular weight (kDa)	No. of injections	Comparison group	Follow up (months)	Pain improvement
HA injections in Hip osteoarthritis											
Vad [58]	2003	Prospective	Hip	22	56.4	Hylan G-F 20^®^	6000	3	None	12	VAS pain improved
Berg [75]	2004	Prospective	Hip	31	NA	Durolane^®^	90,000	1	None	3	Not reported
Caglar-Yagci [76]	2005	Prospective	Hip	14	65.3	Hylan G-F 20^®^	6000	3	None	3	VAS pain improved
Tikiz [77]	2005	RCT	Hip	48	60.4	Hylan G-F 20^®^	6000	3	Na hyaluronate	6	VAS pain improved
Qvistgaard [78]	2006	RCT	Hip	88	66	Hyalgan^®^	730	3	Saline/prednisolone	3	No difference from saline group
Gaston [79]	2007	Prospective	Hip	13	64	Suplasyn	NA	3	None	6	Not reported
Rennesson-Rey [80]	2008	Prospective	Hip	55	NA	Hylan G-F 20^®^	6000	1	None	6	VAS pain improved
Richette et al. [81]	2009	RCT	Hip	85	60	Adant^®^	900	1	Saline	3	VAS pain improved
Migliore et [59]	2009	RCT	Hip	42	70	Hyalubrix^®^	3200	2	Mepivacaine	6	VAS pain improved
Spitzer et al. [82]	2010	RCT	Hip	312	59	Hylan G-F 20^®^	6000	2	Prednisolone		
Eyigör [83]	2010	Prospective	Hip	21	61.3	Adant^®^	900	3	None	6	VAS pain improved
Atchia et al. [84]	2011	RCT	Hip	57	69	Durolane^®^	90,000	1	Saline/prednisolone	2	No difference from saline group
Battaglia et al. [57]	2013	RCT	Hip	100	53	Hyalubrix^®^	3200	3	PRP		VAS pain improved
Dallari et al. [85]	2016	RCT	Hip	80	20–60	Hyalubrix^®^	3200	3	PRP	12	WOMAC pain improved
HA injections in ankle osteoarthritis											
DeGroot et al. [86]	2012	RCT	Ankle	56	54	Supartz	620–1170	1	Saline	3	VAS pain improved
Cohen et al. [87]	2008	RCT	Ankle	28	56.2	Hyalgan^®^	500–730	5	Saline	6	WOMAC pain improved
Salk et al. [88]	2006	RCT	Ankle	17	57.8	Hyalgan^®^	500–730	5	Saline	6	No difference from saline group
Karatosun et al. [89]	2008	RCT	Ankle	30	52.1	Adant^®^	900	3	Physical therapy	12	VAS pain improved
Carpenter [90]	2008	Prospective	Ankle	26	57	Synvisc-one^®^	6000	3	Arthroscopy	13	Pain improved
Sun et al. [91]	2011	Prospective	Ankle	46	51.7	Hyalgan^®^	500–730	3	None	6	Pain improved
Luciani et al. [92]	2008	Prospective	Ankle	21	45	Synvisc-one^®^	6000	3	None	18	VAS pain improved
Witteveen et al. [93]	2008	Prospective	Ankle	55	55	Synvisc-one^®^	6000	1	None	3	VAS pain improved
Sun et al. [94]	2006	Prospective	Ankle	75	50.2	Artz	620–1200	5	None	6	Pain improved
HA injections in knee osteoarthritis											
Grecomoro [95]	1987	RCT	Knee	34	65	Hyalgan^®^	500–730	3	Placebo	1.5	VAS pain improved
Puhl [96]	1993	RCT	Knee	209	61	Supartz	600–1200	5	0.25 mg hyaluronate/2.5 ml	2	VAS pain improved
Henderson [97]	1994	RCT	Knee	91	66	Hyalgan^®^	500–750	5	Placebo	0	No superior effect compared to placebo group (in mean joint space width (JSW) of the medial compartment)
Adams [98]	1995	RCT	Knee	102	61	Hylan G-F 20^®^	6000	3	NSAIDs/Arthrocentesis	2	WOMAC pain improved
Lohmander [99]	1996	RCT	Knee	240	58	Artzal ™	1000	5	Placebo	6	VAS pain improved
Altman [44]	1998	RCT	Knee	495	64	Hyalgan^®^	500–730	5	Placebo/NSAIDs	3	WOMAC pain improved
Huskisson [53]	1999	RCT	Knee	100	65	Hyalgan^®^	500–750	5	Placebo	3	VAS pain improved
Brandt [48]	2001	RCT	Knee	226	66	Orthovisc^®^	1000–2900	3	Placebo	3	VAS pain improved
Bunyaratavej [100]	2001	RCT	Knee	49	59	Hyalgan^®^	500–730	4	Placebo	1.6	VAS pain improved
Jubb [101]	2003	RCT	Knee	408	64	Hyalgan^®^	500–730	3	Placebo	3.5	No superior effect compared to placebo group (in mean joint space width (JSW) of the medial compartment)
Altman [44]	2004	RCT	Knee	347	63	Durolane	1000	1	Placebo	3	No superior effect compared to placebo group
Chevalier [102]	2010	RCT	Knee	235	63.6	Synvisc-one^®^	6000	1	Placebo	6.5	WOMAC pain improved
Huang [55]	2011	RCT	Knee	100	65.9	Hyalgan^®^	500–730	5	Placebo	6.25	WOMAC pain improved
Lundsgaard [45]	2008	RCT	Knee	84	68.8	Hyalgan^®^	500–730	4	Placebo	6.5	No superior effect compared to placebo group
Neustadt [50]	2005	RCT	Knee	115	58.4	Orthovisc^®^	1000–2900	4	Arthrocenteses	5.5	WOMAC pain improved
Çubukçu [51]	2004	RCT	Knee	30	52.6	Hylan G-F 20^®^	6000	8	Placebo	2	WOMAC pain improved
Day [49]	2003	RCT	Knee	116	62	Artz™	620–1200	5	Placebo	4.5	WOMAC pain improved
Petrella [52]	2006	RCT	Knee	51	63.9	HA sodium salt	NA	6	Placebo	3	VAS pain improved
Petrella [103]	2002	RCT	Knee	54	65.5	Suplasyn^®^	NA	3	Placebo	1	VAS pain improved
Strand et al. [104]	2012	RCT	Knee	249	60	Gel-One	NA	1	Placebo	3	WOMAC pain improved
Sun [40]	2017	RCT	Knee	132	62.7	HYA-JOINT Plus	NA	1	Synvisc-One	6	VAS pain improved

For oral HA treatment, the body absorbs the high molecular weight polymer as a decomposed 2–6 membered polysaccharide [29]. One proposed mechanism of action shows that ingested HA binds to Toll-like receptor-4 and promotes the expressions of interleukin-10 and cytokine signaling, which both lead to anti-inflammation of arthritis [30]. a systematic review of 13 reports on oral HA clinical trials, Oe et al. found that patients that were on a highly pure HA regiment reported a beneficial effect on knee pain compared to placebo [23]. In terms of safety, it has been shown that on a 12 month study of 30 patients taking an oral HA regiment, no statistically significant negative side effects were seen [31].

As stated before, locally injected HA differs in many different characteristics. The most fundamental change is the molecular weight of the HA in the injection, and it was shown that there is no significant difference in the long term outcome regardless the preparation [32]. Unlike oral treatment, the complete HA molecule is introduced to the synovial fluid of the affected joint, providing a variety of different mechanisms for symptom relief [33]. These include enhancing the synthesis of extracellular matrix proteins, altering inflammatory mediators in order to shift away from degradation, reducing the motility of lymphocytes, and maintaining cartilage thickness, area and surface smoothness [24]. However, it must be stated that these are not the only proposed mechanism of actions for locally injected HA and further research trials needs to be performed in order to fully investigate the physiological effects of the treatment. Based on the study of 76 trials by Bellamy et al. locally injected HA treatment is an effective treatment for OA based on its effects of patients pain, function and patient global assessment [34]. In terms of safety, it has also been shown that there is no statistically significant negative side effects in patients receiving injection treatment [35].

Studies have shown that both local injections and oral supplementation of HA can combat OA symptoms, especially with those with early osteoarthritis [36]. Interestingly, Panuccio et al. showed that if these two types of treatments are combined, the oral supplementation of HA can extend the benefits of the injection treatments [37]. Thus, patients would not have to visit hospitals and receive the sometimes-uncomfortable injections as often [37]. Further randomized clinical trials are required to be designed in order to determine the exact outcomes of combined treatment.

## Hyaluronic acid based tissue engineering (modified therapy, biomaterial, scaffold and stem cells)

Hyaluronic acid serves as a valuable material to create hydrogels that assist in healing because of its non-immunogenic properties, controlled biodegradability, biocompatible polymerization chemistry and multiple different reaction sites [38]. However, native HA is not useful and must be first cross-linked in order to provide stability and improve functionality of the gels [39]. In order to crosslink HA, different methods such as water-soluble carbodiimide crosslinking, polyvalent hydrazide crosslinking, divinyl sulfone crosslinking, disulfide crosslinking, and photo-crosslinking through glycidyl methacrylate-HA conjugation have been used [40].

Cross-linked HA hydrogels have several applications in the field of bioengineering. These include processes such as cell delivery, molecule delivery, cartilage tissue engineering, and development of micro-device systems [41]. Hydrogel scaffolds can be embedded with mesenchymal stem cells (MSCs) in order to boost the efficacy of regenerative capacity of MSCs [23, 25]. When paired with an HA hydrogel, MSCs have been shown to undergo chondrogenic differentiation, which leads to neo-cartilage formation and recovery of some of the degraded cartilage of patients with OA [26]. However, these MSCs also undergo hypertrophic phenotype changes as an adverse effect of being within the hydrogel scaffold, which lead to extensive calcification of the neo-cartilage matrix [27]. To combat the calcification and hypertrophic changes thus leading towards more chondrogenesis, specific HA hydrogel scaffolds are being engineered that facilitate the latter process and hinder the former processes [27, 28].

The most common molecule paired with HA scaffolds are growth factors, which recently have been shown to be able to recruit endogenous stem cells to a defect site and allow for de novo tissue regeneration [41]. With cartilage tissue engineering, chondrocytes can be encapsulated into hydrogel networks in order to treat the damaged cartilage tissue [41]. Taking advantage of the spatial control of certain types of HA hydrogels, microdevice systems are being developed that can encapsulate viable embryonic stem cells and then retrieved later using mechanical disruption [41]. These stem cells could then be used for treatment of diseases such as OA.

HA hydrogels are sometimes synthesized into scaffolds that can aid in the creation of new tissue [42]. In general, the scaffolds’ modes of action are dependent on their physical properties, mass transport properties, and biological properties [42]. Specifically for HA, those stated properties are dependent on things such as the molecular weight of HA, whether HA is composited with another polymer, degree of grafting, crosslinker type and crosslink densities, as well as interaction with cell surface receptors [42]. With all these mechanisms in mind, it’s easy to see how valuable HA is for creating scaffolds because of its natural properties that can be modified in a variety of different ways.

## Human clinical studies involving hyaluronic acid

Improved hydrogels are continuously being made that seek to maximize the effect of treatment. One such is called Gel-One, which is composed of a product called Gel-200, a cross-linked hyaluronate hydrogel [43]. This product was first shown to produce chondroprotective, anti-inflammatory effects and long-lasting analgesia in OA mouse models [43]. Another new product, HYA-JOINT Plus, was shown to produce a longer lasting and stronger effect on pain than compared to Synvisc-one, which is currently used by many physicians in intra-articular injections [44]. Furthermore, the product Cingal combined HA hydrogels with triamcinolone hexacetonide, a long acting corticosteroid previously shown to help with arthritis [45]. A clinical trial showed that Cingal provided immediate and long-term relief of osteoarthritis-related pain, stiffness, and function through 26 weeks when compared to saline [45]. Additionally, a new product named Cartistem paired HA hydrogels with human umbilical cord blood-derived mesenchymal stem cells has been used [46]. Unlike the usual autologous MSCs, these allogenic stem cells are isolated in a noninvasive manner and have shown a high expansion capacity, allowing a plethora of cells for therapeutic applications [46]. Moreover, it was previously shown in a sheep model of osteoarthritis that allogenic MSCs have a similar efficacy of treatment as autologous MSCs [34]. In a clinical trial, the product showed maturing repair tissue at 12 weeks and pain lowered at 24 weeks, both of which remained stable over 7 years of follow-up [33].

Over the last decades, several clinical trials have been developing many HA preparations and investigating their efficacy and safety. Although many studies have demonstrated that the use of intra-articular HA injection would be beneficial, non-surgical option for OA and may delay the need for joint replacement [47]. However, there is an ongoing controversy over the clinical effectiveness and sustainability of intra-articular injection of HA for OA patients. In 2004, Altman et al. [48] concluded that Durolane^®^ (a non-animal stabilized product with very high molecular weight HA; 100,000 kDa) [49] is not beneficial and had no superiority over the placebo treated groups in terms of The Western Ontario and McMaster Universities Osteoarthritis Index (WOMAC) score and other efficacy parameters. In addition, Lundsgaard et al. [50] demonstrated that there is not any significant difference between the intra-articular injection of HA (Hylagan^®^; 6000 kDa) and injection of physiological saline in patient with knee OA.

The influence of HA molecular mass on the clinical and functional efficacy remains debatable. In 2005, Karatosun and his colleagues [51] reported that there is no statistically significant difference between intra-articular injections of both high and low molecular weight hyaluronic acid for late-stage knee OA patients. Both groups experienced a substantial improvement in the outcome parameters at the latest follow-up. On the other hand, Berenbaum et al. [1] demonstrated that 3 weekly injections of GO-ON (intermediate MW HA; 800–1500 kDa) had statistical superiority (95% CI all > 0, p = 0.021) over Hyalgan^®^ (Low MW HA; 500–730 kDa) for knee OA symptoms over 6 months.

Many recent trials and meta-analyses have evaluated and proved the substantial superiority of injectable HA over the placebo group for pain relief and functional efficacy [52]. The clinical trial performed by Brandt et al. [48] and Day et al. [49] indicated that HA is safe and well-tolerated to induce a clinically significant improvement for patients with mild-to moderate knee OA. Furthermore, Neustadt et al. [53] reported that HMW-HA (Orthovisc^®^) also improve clinical outcome in advanced stage of OA (K-L grade 4).

One of the main controversial issues in this field is the timing and duration of injection and whether it may have an impact on its efficacy and sustainability. Cubukuc et al. [51] compared the intra-articular 3 weekly injections of Hylan G-F 20 and saline in OA patients. They reported that the optimal pain relief was noticed in HA group as early as 3rd week while functional improvement was seen at 8th week. In 2006, Patrella et al. [54] designed randomized controlled trial to determine the difference between three versus 6 consecutive weekly HA injections. They demonstrated that there are no differences between 3 and 6 HA injections in regard of pain, function, and patient satisfaction. In 1999, Huskisson et al. [53] demonstrated that 5 weekly intra-articular injections of sodium hyaluronate (Hyalgan A) would provide a symptomatic improvement which persisted for 6 months. Recently, two large, controlled randomized clinical trial confirms that 5 weekly IA injections of HA (Hyalgan) in patients with knee OA provided sustained relief of pain and improved patient function, and were at least as effective with fewer adverse reactions as continuous treatment with naproxen for 26 weeks [55, 56]. From the cost-effectiveness stem point, Hyalgan^®^ and Supartz^®^ are considered as economically feasible to provide a rapid pain relief and functional outcomes when compared to Orthovisc^®^ and Synvisc^®^. Despite the more number of injections required in Hyalgan^®^ and Supartz^®^ courses, it still costs healthcare plans less than Synvisc^®^ [57].

Many trials have been performed to investigate the role of intra-articular injection of HA in alleviating the symptoms in hip osteoarthritis. A randomized clinical trial compared HA injection to platelet rich plasma (PRP) injection, Battaglia et al. [58] demonstrated that HA is superior to PRP in patients with symptomatic hip OA in term of pain relief and functional improvement. In 2003, Vad et al. **[**59**]** reported that three HA injections would be safe and an essential option for mild-to moderate hip OA to produce a rapid pain relief. However, they showed no efficacy for the patients with severe hip OA. Apart from that Intra-articular injection of low and high molecular weight HA was notably effective in relieving the pain, it was also associated with reduction of 48.2% of NSAIDs consumption at the 3rd month when compared with baseline values [60]. The conclusion of meta-analysis of 26 clinical trials indicated that HA injection would be consider as the best conservative line for hip OA with substantial pain relief and function amelioration. However, there is no clear evidence to prove its ability to modify the morphological or radiological changes of the pathological hip [61].

A recent meta-analysis including nine clinical trials exploring the effectiveness of intra-articular HA injection for the treatment of ankle OA and to investigate the effects of modified regimens of HA. Its results suggest that intra-articular HA administration can significantly reduce pain for patients with ankle OA. In addition, Intra-articular HA was likely superior to other conservative therapy. They suggested the use of multiple doses with an appropriate injection volume would achieve maximum therapeutic effects [62].

In 2013, a pilot study examined the effectiveness of the intra-articular injection of Euflexxa in 22 patients with osteoarthritis of the subtalar joint [63]. Euflexxa is a bioengineered 1% sodium HA that does not require cross-linking, has a molecular weight range of 2.4–3.6 mDa and is synthesized via controlled fermentation [55]. The study showed that patients significantly experienced an improvement in American Orthopedic Foot and Ankle Society Ankle Hind foot scores and visual analog scale assessment as well as longer tolerated walking distance [63]. Likewise, a clinical trial was conducted to explore the effectiveness and safety of the HA-chondroitin sulfate medication Arthrum HCS [64]. Arthrum HCS is a mixture of 40 mg hyaluronic acid and 40 mg chondroitin sulfate in a 2-mL solution [64]. They investigated one hundred and twelve patients and showed that Arthrum lowered the WOMAC sub score A in assessments at 1, 3 and 6-month intervals after treatment [64]. Additionally, the trial showed that patients had improved mobility and reduced their consumption of analgesics [64]. In addition, Xin et al. designed a clinical study to compare the efficacy and safety of two different sodium hyaluronate drugs, Adant^®^ and Artz^®^ [65]. The main difference between the two drugs is the manufacturing process, with Adant^®^ being manufactured by fermentation and Artz^®^ being manufactured by extraction of cockscomb [65]. The study concluded that both drugs showed a significant reduction in VAS scores while not showing a significant difference in efficacy and safety [65]. Moreover, another clinical trial was conducted to see the safety and efficacy of Hyalubrix via an observational study of normal medical practice [66]. Hyalubrix is a sterile, nonpyrogenic solution of HA sodium salt with a molecular-weight around 1500 kDa that is produced by bacterial fermentation [67]. The study showed that there was a statistically significant improvement in VAS, HAQ and EuroQol scores while only showing a 0.8% adverse event rate [66].

## Animal studies and future directions for the clinical use of hyaluronic acid

As with all novel disease treatments, animal studies are continuously being designed in order to determine the safety and efficacy of treatment. Some examples for osteoarthritis animal studies include a novel treatment that combines HA, chondroitin sulfate and glucosamine (HA-CSNAG) [68]. Sükür et al. used a rat model with early stage OA and showed that this novel compound (HA-CSNAG) provided a more chondroprotective effect in the rats’ cartilage when compared to HA treatment alone [68]. In 2016, Tamura et al. examined a novel conjugate composed of HA and methotrexate called DK226 [69]. Methotrexate has been one of the most widely used medications for rheumatoid arthritis and has recently been shown to help in osteoarthritis of the knee [69]. Their results showed that the intra-articular injection of DK226 showed similar anti-arthritic effects as oral methotrexate but eliminated the harmful side-effects that usually come with oral treatment [69].

A study performed by Ishikawa et al. to investigate the biocompatibility and adverse effects of the aforementioned medication Gel-200 [70]. They used subcutaneous air pouch rat model as well as knee joints of normal rabbits [70]. They concluded with showing that Gel-200 did not induce any granulomatous inflammation nor a significant thickening of the fibrous belt often seen in the air pouch models [70]. This was compared to the hylan G-F 20 medication Synvisc which did the exact opposite and showed a granulomatous inflammation and thickening of the fibrous belt [70].

Additionally, nanoparticles composed of poly (d,l-lactic acid) (PLA) or poly(d,l-lactic-*co*-glycolic acid) (PLGA) covered by chemically esterified amphiphilic HA are being considered as drug carriers for treatment of OA [71]. A study using healthy rat models was designed to examine if the nanoparticles had a toxic effect on the model knees [71]. Zille et al. showed that the nanoparticles did not modify the synovial membranes and did not upregulate the cytokines IL-1β and TNF-α [71]. A separate study was done using 350 g female Sprague- Dawley rats to study if the nanoparticles were retained in the joint and concluded that in 70% of the rats, the nanoparticles were retained for at least a week [72].

## Strengths and limitations of hyaluronic acid treatment

As stated before, HA treatment has shown many beneficial effects in studies and experiments such as intraarticular lubrication, anti-inflammatory, analgesic and chondroprotective effects, among others [20]. However, considering its cost it is not always the recommended treatment for OA patients. According to both the Osteoarthritis Research Society International 2012 guideline and the American College of Rheumatology 2013 guidelines, HA treatment is neither recommended or discouraged because of the inconsistency of clinical studies [73, 74]. For a plethora of the studies both societies researched, a large placebo effect was seen which limited the scope of the data [73, 74]. The treatment also does not provide an immediate relief to most patients, as studies have shown that it takes about 5 weeks before patients feel the full effect of the treatment [75]. Despite the demonstrated efficacy and the safety of HA products, there are few associated side effects that mostly limited to local pain and swelling with frequent injections [76]. Further investigations are still required to obtain specially designated molecular mass HA to optimize the clinical effect and extend its applications. In addition, more randomized controlled trials with a large sample to test not only the efficacy of HA versus the other established therapies of OA, but also the different products, dosages and the optimal number of injections.

## Conclusion

Osteoarthritis is a debilitating disease that affects a large portion of the population. As the general age of the population continues towards an older age, the prevalence of the disease is only going to go up. Therefore, more research is needed in order to fully control the disease and its side effects. Hyaluronic acid is a potential bright spot for helping lower the side effects. Its effectiveness is due to the many methods of actions it deploys, including lubrication, anti-inflammatory and chondroprotective effects. Treatment can be done both orally and through intra-articular injections. New products are continuously being developed that change the composition of the molecule as well as pairing it with other drugs to maximize the effect. Hyaluronic acid treatment shows a lot of potential that will hopefully be discovered through continued research.
